# Perceived Overqualification and Intensive Smartphone Use: A Moderated Mediation Model

**DOI:** 10.3389/fpsyg.2022.794913

**Published:** 2022-02-25

**Authors:** Xiongliang Peng, Kun Yu, Kairui Zhang, Hanbing Xue, Jianfeng Peng

**Affiliations:** Renmin University of China, Beijing, China

**Keywords:** intensive smartphone use, perceived overqualification, affective commitment, job boredom, person-environment (PE) fit theory

## Abstract

Previous studies only considered the impact of personal or environmental factors on intensive smartphone use separately, while largely ignoring the impact of person-environment (P-E) fit on it. Drawing on the P-E fit theory, we proposed that perceived overqualification (POQ), an indicator of person-job misfit, positively affects intensive smartphone use *via* job boredom, and affective commitment moderates this indirect effect. We examined our hypotheses using four-wave time-lag data of 450 workers from 62 teams. The results revealed that POQ raised job boredom of an individual and thus increased their intensive smartphone use. In addition, when the affective commitment was high, the indirect effect from POQ to intensive smartphone use *via* job boredom was weaker. The implications, limitations, and future directions of this research were discussed.

## Introduction

Smartphones, which can be used for making phone calls, bring many conveniences to people and are already in widespread use (Spagnoli et al., 2019; Busch and McCarthy, 2021). However, intensive use of smartphones may be problematic and trigger adverse consequences, such as depression (Harwood et al., 2014; Kliestik et al., 2020; Lǎzǎroiu et al., 2020), anxiety (Hartanto and Yang, 2016; Green et al., 2020; Taylor et al., 2020), and wellbeing (David et al., 2018). Considering the potential negative consequences of intensive smartphone use, increasing attention has been paid to its formation mechanism, which could provide substantive suggestions on how to avoid those adverse outcomes (Busch and McCarthy, 2021). Previous literature on the antecedents of intensive smartphone use mainly includes personal characteristics, such as self-control (Berger et al., 2018; Servidio, 2019), emotional instability (Roberts et al., 2015), and self-efficacy (Chiu, 2014), and environmental factors, such as dependence on the smartphone for work (Li and Lin, 2018), conformity (Chen et al., 2017), and perceived stress (Liu et al., 2018).

However, previous studies either considered the impact of personal or environmental factors on intensive smartphone use separately, while ignoring the possible interactions between personal and environmental factors, such as person-environment (P-E) fit. The fit between person and environment brings individuals pleasant emotions and positive motivations, which, in turn, leads to a series of positive consequences, such as job self-efficacy (Yu and Davis, 2016), creativity (Luksyte and Spitzmueller, 2016), and voice behavior (Erdogan et al., 2020). The P-E fit theory posits that there are five types of P-E fit, in which person-job fit is particularly important for individuals, especially in the workplace (Kristof-Brown et al., 2005; Jansen and Kristof-Brown, 2006; van Vianen, 2018). For instance, perceived overqualification (POQ), a typical type of person-job misfit in organizations (Erdogan et al., 2020) and a global trending phenomenon (Hu et al., 2015; Li et al., 2021; Zhang et al., 2021a), was proved from different theoretical perspectives that could rise many negative consequences in the workplace (Feldman, 1996; Harari et al., 2017), such as more counterproductive behavior (Liu et al., 2015; Schreurs et al., 2020), high turnover (Wu and Chi, 2020), less creative performance (Zhang et al., 2021b), and less proactive behavior (Luksyte et al., 2020). Furthermore, recent research has found that POQ leads to more cyberloafing in the organization (Cheng et al., 2020). Compared with using computers to cyberloafing on the Internet, due to the convenience of the smartphone, more and more employees deliberately use smartphones intensively during working hours (Busch and McCarthy, 2021). Unfortunately, the investigation on whether POQ has an association with intensive smartphone use, and if so, how and when, is largely absent from the literature.

The P-E fit theory states that when individuals perceive misfits between a person and the job, they may experience a type of negative emotion called job boredom (Edwards and Van Harrison, 1993; Kim et al., 2021). Job boredom is defined as an unpleasant and deactivated emotion, which is characterized by low arousal and dissatisfaction caused by a lack of a stimulating work environment (Reijseger et al., 2013). Given that boredom is an emotional response to a less stimulating work environment, it is usually experienced by overqualified employees (Liu and Wang, 2012; Sánchez-Cardona et al., 2020; Kim et al., 2021). Furthermore, the P-E fit theory also suggests that individuals could take some measures to cope with the negative emotions caused by person-job misfits (Kristof-Brown et al., 2005; van Vianen, 2018). For instance, empirical research has shown that job boredom could promote individuals to use smartphones frequently (Smetaniuk, 2014; Fullwood et al., 2017). Therefore, we proposed that job boredom may be a critical mechanism to explain the relationship between the POQ of employees and their intensive use of smartphones.

Additionally, the P-E fit theory proposes that different types of fit could complement or strengthen each other (Kristof-Brown et al., 2005; van Vianen, 2018). Therefore, the relationship between person-job misfit (POQ) and job boredom may be affected by other types of fit. For instance, the affective commitment of employees to the organization, as a typical person-organization fit, may alleviate the adverse impact of the person-job misfit (van Vianen, 2018). In fact, previous empirical studies have provided preliminary evidence for the mitigating effect of person-organization fit on other misfits (Erdogan et al., 2020; Zhang et al., 2021b).

**FIGURE 1:**
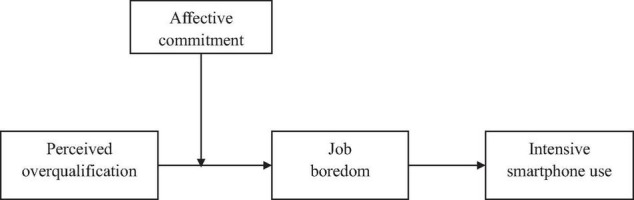
Proposed research model.

Therefore, the purpose of this research was to investigate the relationship between POQ and intensive smartphone use, its underlying mechanism, and the boundary conditions. According to the P-E fit theory (Kristof-Brown et al., 2005; Edwards, 2008), we investigated the influence of POQ on intensive smartphone use through the mediation of job boredom. Moreover, in response to the impact of different fit interactions on employees (Oh et al., 2014; Harold et al., 2016), we assumed that affective commitment alleviates the positive impact of POQ on job boredom. Finally, we proposed that affective commitment buffers the indirect effect of POQ on intensive smartphone use *via* job boredom. Our moderated mediation model is depicted in Figure 1.

Two contributions of this study to the literature are worth noting. First, drawing on the P-E fit theory (Kristof-Brown et al., 2005; Edwards, 2008; van Vianen, 2018), this study enriches our understanding of the antecedents of intensive smartphone use from an interactive perspective of person and environment and helps us better understand how POQ leads to intensive smartphone use through an emotional route. Second, by examining the interactive effect of affective commitment, an indicator of person-organization fit, and POQ on job boredom and intensive smartphone use, this study addresses the issue of inconsistent conclusions in intensive smartphone use literature due to the lack of exploration of boundary conditions (Busch and McCarthy, 2021) and well answers the scholastic call for the interactions between different fitting types (Kristof-Brown et al., 2005; Jansen and Kristof-Brown, 2006).

## Hypothesis Development

### The Mediating Role of Job Boredom

The P-E fit theory offers a solid theoretical foundation for studying the impact of POQ on intensive smartphone use. According to the P-E fit theory (Edwards, 2008; van Vianen, 2018), when individuals are compatible (incompatible) with one or more characteristics of the work environment, fit (misfit) will occur and thus produce positive (negative) results for the individuals and organizations. The characteristic of the work environment mainly includes job, organization, vocation, team, and supervisor (Jansen and Kristof-Brown, 2006). Therefore, in terms of content, the P-E fit includes five types (van Vianen, 2018). Among them, person-job fit means that individual abilities and job demands and supplies are supposed to be fit appropriately (Edwards, 2008; Rodrigues et al., 2020). Based on this definition, POQ, which refers to the perception of individuals of their qualifications, such as knowledge, skills, abilities (KSAs), work experience, and education beyond the job requirements (Erdogan and Bauer, 2009; Chen et al., 2021; Zhang et al., 2021c), is a typical person-job misfit (Liu and Wang, 2012). Previous studies have found that POQ could bring several adverse consequences to employees (Harari et al., 2017), such as more cyberloafing (Cheng et al., 2020), high turnover (Wu and Chi, 2020), and less proactive behavior (Luksyte et al., 2020).

However, although the effect of job boredom, an unpleasant state commonly links to insufficient stimulation in the work environment (Mikulas and Vodanovich, 1993; Reijseger et al., 2013), on intensive smartphone use was well documented in the literature (Elhai et al., 2018; Wang et al., 2020), and there are relatively few studies on the association between POQ or other forms of person-job misfit and job boredom (Harju and Hakanen, 2016; Sánchez-Cardona et al., 2020). For the following reasons, we proposed that POQ could affect intensive smartphone use through job boredom.

First, the KSAs of overqualified employees far go beyond the job requirements, which indicates that the stimulation provided by the job is not enough to attract the interests of employees. The misfit between person and job will result in the boredom of employees (Reijseger et al., 2013; Harju and Hakanen, 2016). In fact, prior studies have provided preliminary evidence for the effect of POQ on job boredom (Harju and Hakanen, 2016; Sánchez-Cardona et al., 2020). Second, when individuals feel bored at work, they usually try to deal with it by distracting themselves (e.g., intensive smartphone use) rather than focusing on work tasks (Reijseger et al., 2013). As mentioned above, previous literature has given sufficient evidence on the effect of job boredom on intensive smartphone use (Elhai et al., 2018; Wang et al., 2020). In summary, overqualified individuals are likely to experience job boredom due to their qualifications exceeding the job requirements, and they will more likely to use the smartphone intensively to eliminate the discomfort caused by this misfit and boredom. Accordingly, we proposed the following hypothesis:

*H1: Job boredom mediates the relationship between POQ and intensive smartphone use.*

### The Moderating Role of Affective Commitment

According to the P-E fit theory (Edwards, 2008; van Vianen, 2018), among the five types of P-E fit, the person-organization fit is the most studied one (Kristof-Brown et al., 2005; Jansen and Kristof-Brown, 2006) and mainly refers to an individual identifying with the value of the organization and having a sense of dependence on the organization (van Vianen, 2018). Based on this definition, affective commitment, as the core of organizational commitment, means identification of an individual with, participation in, emotional attachment to an organization and willingness to be a member of the organization (Meyer et al., 1993; Lin, 2010; Taylor et al., 2012; Vandenberghe et al., 2017), can be categorized as a type of person-organization fit (Kristof-Brown et al., 2005; Jansen and Kristof-Brown, 2006).

Based on the P-E fit theory (Edwards, 2008; van Vianen, 2018), the impact of person-job fit on the outcomes is most likely to be moderated by person-organization fit (Kristof-Brown et al., 2005; Erdogan et al., 2020). Along with this logic, the consequences of person-job misfit on job boredom and intensive smartphone use may be alleviated by person-organization fit too. Thus, affective commitment, as a type of person-organization fit, is likely to alleviate the job boredom caused by POQ (person-job misfit). For instance, overqualified individuals with a high affective commitment have a strong emotional attachment to the organization (Meyer et al., 1993). They may reduce the job boredom caused by the person-job misfit by shifting their attention to the organization (Harju and Hakanen, 2016). Therefore, we proposed that affective commitment could alleviate the job boredom caused by POQ and also weaken the mediation path from POQ to intensive smartphone use *via* job boredom. To sum up, we assumed the following hypotheses:

*H2: Affective commitment moderates the positive impact of POQ on job boredom. Specifically, the positive effect is weaker when affective commitment is higher.*

*H3: Affective commitment moderates the mediating effect of job boredom in the relationship between POQ and intensive smartphone use. Specifically, the mediating effect is weaker when affective commitment is higher.*

## Materials and Methods

### Sample and Procedures

We surveyed data from a large state-owned enterprise in China, which is part of a larger data collection. The enterprise was mainly responsible for maritime traffic and transportation, so all employees were men and belonged to several teams. After contacting the top management of the enterprise, they expressed great willingness to cooperate and appointed a human resources staff to be responsible for the research. Before the questionnaire survey, we told team leaders and employees that the survey was only for academic research and there was no reward. They could freely decide whether to participate in the survey or not. Finally, with the help of the staff in charge, we set up a WeChat (an instant messaging app) group for each team willing to participate in the research and distributed questionnaires *via* those online groups.

All variables were self-reported, which might lead to common method bias (CMB; Podsakoff et al., 2003). For the sake of reducing the impact of CMB on the research results, data collection was carried out in four stages, each with an interval of 2 weeks. Specifically, POQ, age, job tenure, and education were measured at Time 1; affective commitment was measured at Time 2; job boredom was measured at Time 3; and intensive smartphone use was measured at Time 4. All participating employees were full-time men, and we ensured them that their responses were anonymous and confidential.

When we initially contacted all 530 employees (67 teams) of the organization to ask for participation, we got an 89.8% response rate. Considering the sample fitting, only the responses of all employees collected from the four waves of data were included in the final analysis. The final sample was 450 employees (62 teams), and the response rate was 84.9%. The average age of all participants was 35.36 years (*SD* = 7.57), and the average tenure was 13.00. Moreover, 47.1% of the participants had a bachelor’s degree or above.

### Measures

According to the translation-back-translation standard, we translated all the English items into Chinese (Brislin, 1980). All the items described below were surveyed with the seven-point Likert scale unless specifically noted.

### Perceived Overqualification

We measured POQ with nine items using a scale adapted from Maynard et al. (2006), which was commonly used in the POQ literature (Erdogan et al., 2020; Wu and Chi, 2020). A sample item was “I have job skills that are not required for this job” (α = 0.82).

### Affective Commitment

We measured affective commitment using a six-item scale developed by Meyer et al. (1993). An example of the item was “This organization has a great deal of personal meaning for me” (α = 0.77).

### Job Boredom

We measured job boredom using a six-item scale (Reijseger et al., 2013). A sample item was “At my work, there is not so much to do” (α = 0.92).

### Intensive Smartphone Use

A three-item scale developed by Spagnoli et al. (2019) was used to gauge intensive smartphone use. A sample item was “At my work, there is not so much to do” (α = 0.86).

### Control Variables

According to the literature on POQ and smartphone use, we controlled age, education level, and job tenure of employees (Kwon et al., 2016; Zhang et al., 2016; Barnes et al., 2019; Busch and McCarthy, 2021).

## Results

### Preliminary Analyses

The descriptive statistics and correlations between variables are exhibited in Table 1. As shown in Table 1, POQ was positively related to job boredom (*r* = 0.21, *p* < 0.01) and intensive smartphone use (*r* = 0.13, *p* < 0.01). Job boredom was positively related to intensive smartphone use (*r* = 0.17, *p* < 0.01).

**TABLE 1:** Descriptive statistics and correlations between variables.

	*M*	*SD*	1	2	3	4	5	6	7
1. Age	35.36	7.57	–						
2. Education	1.49	0.54	–0.19**	–					
3. Job tenure	13.00	8.16	0.95**	–0.24**	–				
4. POQ	3.82	1.04	–0.09	0.18**	–0.13**	(0.82)			
5. Affective commitment	4.82	1.00	0.01	–0.02	0.02	–0.20**	(0.77)		
6. Job boredom	2.52	1.26	–0.10*	0.09	–0.10*	0.21**	–0.41**	(0.92)	
7. Intensive smartphone use	3.39	1.12	–0.10*	0.08	–0.07	0.13**	–0.05	0.17**	(0.86)

N = 450. For education, 1, college degree and below; 2, undergraduate course; 3, master degree or above. Reliabilities are on the diagonal. *p < 0.05. **p < 0.01.

Since all variables were self-reported by employees (i.e., POQ, affective commitment, job boredom, and intensive smartphone use), confirmatory factor analyses (CFA) were performed using Mplus 8.3 to test the discrimination validity of variables. Considering the number of observed indicators, we adopted the random packing method to pack the items, which avoids non-convergence issues and improves the reliability of indicators (Hall et al., 1999; Nasser and Wisenbaker, 2003). POQ, affective commitment, and job boredom were all randomly grouped into three items. As demonstrated in Table 2, the hypothesized four-factor model fitted better to data [χ^2^(48) = 83.05, *p* < 0.001, CFI = 0.99, TLI = 0.98, RMSEA = 0.04, SRMR = 0.04] than alternative models. The results of CFA demonstrated that our variables were well differentiated.

**TABLE 2:** The results of confirmatory factor analysis (*N* = 450).

Model	χ^2^	df	χ^2^/df	TLI	CFI	SRMR	RMSEA
Four-factor model: Proposed structure	83.045**	48	1.73	0.98	0.99	0.04	0.04
Three-factor model: Combining W and M	322.263**	51	6.32	0.86	0.89	0.07	0.11
Three-factor model: Combining X and W	438.103**	51	8.59	0.80	0.84	0.11	0.13
Two-factor model: Combining X, M, and W	754.189**	53	14.23	0.64	0.72	0.13	0.17
One-factor model: Combining all variables	1382.414**	54	25.60	0.34	0.46	0.17	0.23

TFI, Tucker-Lewis index; CFI, comparative fit index; RMSEA, root mean square error of approximation; SRMR, standardized root mean square residual. X, perceived overqualification; W, affective commitment; M, job boredom; Y, intensive smartphone use. **p < 0.01.

### Analytical Strategy

Considering the nested nature of the data, we conducted a one-way random ANOVA of the outcome variable (intensive smartphone use) to determine whether the multilevel analysis is required. The results revealed that variance in intensive smartphone use was significant [*F*(61, 388) = 1.59, *p* < 0.01], and the intra-class correlation (ICC 1) was 0.08, which manifested a clear nest structure. Therefore, we performed multilevel analysis using Mplus 8.3 to estimate the research model.

First, we tested the simple mediation model (Hypothesis 1) by specifying intensive smartphone use as the dependent variable. When testing the mediating effect, we used the original data directly to test the main effect of Level 1 predictors, which was considered reasonable (Hofmann, 1997). Second, in testing the moderation model (Hypotheses 2 and 3), as POQ and affective commitment were both at Level 1, they were group-mean centered to analyze the moderation effect (Enders and Tofighi, 2007). The moderation effect was further elaborated through simple slope analyses (Aiken and West, 1991). To test the moderated mediation effect (Hypothesis 3), we calculated the indirect effect of POQ on intensive smartphone use through job boredom when the affective commitment was above and below 1 *SD*, respectively (Edwards and Lambert, 2007).

### Hypothesis Testing

For the test of Hypothesis 1, we analyzed the simple mediation model, and all results are shown in Table 3. POQ was significantly and positively associated with job boredom (*B* = 0.24, *p* < 0.01), which in turns lead to more intensive smartphone use (*B* = 0.12, *p* < 0.01). The indirect impact of POQ on intensive smartphone use *via* job boredom was 0.03, 95% CI = [0.01, 0.05]. Thus, Hypothesis 1 was supported.

**TABLE 3:** Results for the moderated mediation model.

variables	JO								ISU							
	Model 1				Model 2				Model 1				Model 2			
	B	SE	95% CI		B	SE	95% CI		B	SE	95% CI		B	SE	95% CI	
Age	–0.01	0.02	–0.01	0.02	–0.03	0.03	–0.07	0.04	–0.06*	0.02	–0.10	–0.03	–0.06**	0.03	–0.11	–0.02
Education	0.08	0.12	0.10	0.12	–0.07	0.09	–0.07	0.27	0.12	0.10	–0.12	0.29	0.13	0.10	–0.08	0.32
Job tenure	0.00	0.02	0.00	0.02	0.02	0.03	–0.04	0.05	0.05**	0.02	0.02	0.09	0.05**	0.02	0.01	0.10
POQ	0.24**	0.06	0.15**	0.06	0.11*	0.05	0.02	0.21	0.11*	0.05	0.01	0.18	0.08	0.06	–0.03	0.18
Job boredom									0.12**	0.04	0.05	0.20	0.15**	0.04	0.05	0.22
Affect commitment					–0.48**	0.07	–0.63	0.36					0.02	0.06	–0.07	0.13
POQ × Affect commitment					–0.16**	0.06	–0.25	–0.01					0.06	0.06	–0.06	0.17
*R* ^2^	0.06**				0.17**				0.20**				0.07**			

POQ, perceived overqualification; JO, job boredom; ISU, intensive smartphone use. The p-values are one-tailed; *p < 0.05. **p < 0.01.

**FIGURE 2:**
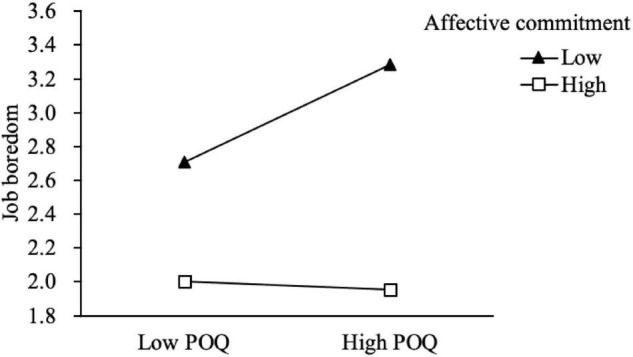
Moderating effect of affective commitment on the relationship between perceived overqualification (POQ) and job boredom.

Hypothesis 2 proposed that affective commitment weakened the positive impact of POQ on job boredom. The interaction effect (Model 2 in Table 3) of POQ and affective commitment on job boredom were negative (*B* = –0.16, *p* < 0.01). Moreover, we plotted the moderating effect of affective commitment in Figure 2 (Aiken and West, 1991). Figure 2 displays that POQ had a weaker positive effect on job boredom when affective commitment was high (+ 1 *SD*; *B* = –0.03, *p* = 0.30) than low (–1 *SD*; *B* = 0.27, *p* < 0.01), thus supporting Hypothesis 2.

Hypothesis 3 posited the moderated mediation effect, in which the indirect effect of POQ on intensive smartphone use *via* job boredom was moderated by affective commitment. As presented in Table 4, compared with low affective commitment (indirect effect = 0.04, *p* < 0.01), when affective commitment was high (indirect effect = 0.00, *p* = 0.30), the indirect effect of POQ on intensive smartphone use was weaker. The difference of indirect effect between high and low affective commitment was –0.04, 95% CI = [–0.09, 0.00]. Thus, Hypothesis 3 was supported.

**TABLE 4:** Conditional indirect effect as a function of affective commitment.

Value of affective commitment	B	SE	95% CI	
–1 *SD* (–1.00)	0.04**	0.02	0.01	0.08
+ 1 *SD* (1.00)	0.00	0.01	–0.03	0.02
Difference	–0.04*	0.02	–0.09	0.00

N = 450. The p-values are one-tailed; *p < 0.05. **p < 0.01.

## Discussion

We used the P-E fit theory (Kristof-Brown et al., 2005; Edwards, 2008; van Vianen, 2018) to investigate the mechanism and boundary conditions of the relationship between POQ and intensive smartphone use in this study. Specifically, we collected four-wave time-lag data of 450 employees from 62 teams to test whether job boredom mediates the relationship between POQ and intensive smartphone use, and how the affective commitment moderates the indirect relationship. Consistent with the P-E fit theory (Kristof-Brown et al., 2005; Edwards, 2008; van Vianen, 2018), we found that job boredom mediates the relationship between POQ and intensive smartphone use. Moreover, affective commitment buffered the mediating effect of job boredom in the relationship between POQ and intensive smartphone use.

### Theoretical Implications

This research contributes to the literature in the following two ways. First, we investigated the formation process of intensive use from a novel lens. A recent review (Busch and McCarthy, 2021) pointed out that previous studies on the antecedents of intensive smartphone use either focused on personal factors or environmental factors separately, ignoring the possible critical role of the P-E fit. The investigation was not comprehensive because individual behaviors are also affected by continuous interactions between individuals and environments (van Vianen, 2018). In addition, previous literature on the antecedents of intensive smartphone use has not conducted in-depth research on the formation mechanism of why individuals use smartphones frequently (Horwood and Anglim, 2019; Porter et al., 2020; Scott et al., 2020). Based on the P-E fit theory (Kristof-Brown et al., 2005; Edwards, 2008; van Vianen, 2018), the examination of POQ and the effect of job boredom on intensive smartphone use enrich the research on the antecedents and formation mechanism of intensive smartphone use from a more interactive perspective.

Second, we further contributed to the literature by answering when POQ would have an impact on job boredom and intensive smartphone use. Previous studies on the antecedents of intensive smartphone use have not reached a consistent conclusion (Busch and McCarthy, 2021), which may be due to the lack of exploration of boundary conditions (Servidio, 2019; Taylor et al., 2020; Wang et al., 2020). Research has shown that job crafting or meaningful work could alleviate the impact of POQ on job boredom (Sánchez-Cardona et al., 2020) while ignoring the impact of the interaction of different fits/misfits on employees, which is urgently called for an investigation (Kristof-Brown et al., 2002; Jansen and Kristof-Brown, 2006). Based on the P-E fit theory (Kristof-Brown et al., 2005; van Vianen, 2018), this study explores the complementary effect of person-organization fit (affective commitment) and person-job misfit (POQ) on job boredom and intensive smartphone use, which also enriches our understanding of the joint effect among different (mis) fits within the P-E fit framework.

### Practical Implications

This study also has two important practical implications. First, considering the harm of the intensive use of smartphones, some scholars put forward suggestions to prohibit the use of smartphones in the workplace (Busch and McCarthy, 2021). This research reveals that overqualified individuals frequently use smartphones due to job boredom. Therefore, reducing the boredom of employees or even preventing them from being bored is the key to the problem of the overuse of smartphones. Otherwise, even if the use of smartphones is prohibited, employees will kill boredom in other ways (e.g., cyberloafing, doodling, and fidgeting). Given overqualified individuals are more likely to experience job boredom (Kim et al., 2021), organizations should select qualified employees rather than overqualified ones. For existing overqualified employees, the organization should assign them more challenging tasks (Kim et al., 2021) to improve their person-job fit and to decrease their job boredom. In addition, previous studies have shown that having more job resources could reduce the job boredom of employees (Harju et al., 2016, 2018; Toscanelli et al., 2021). For instance, giving job autonomy for overqualified employees (Toscanelli et al., 2021) or spaces for job crafting (Harju et al., 2016) could provide them with needed job resources (van Hooff and van Hooft, 2014) and reduce their job boredom, and, finally, lower their frequency of using smartphones.

Second, this study shows that affective commitment reduced the intensive use of the smartphone for overqualified employees. Thus, for those overqualified employees to lower their job boredom and intensive use of smartphones, the organization should carry out strategies to improve their affective commitment, such as providing organizational support (Stinglhamber and Vandenberghe, 2003), reducing their work-family conflict (Wayne et al., 2013), and supervisory mentoring (Lapointe and Vandenberghe, 2017).

### Limitations and Future Directions

Although our research has several contributions, there are still some deficiencies that need to be further improved in the future. First, although we identified the mechanism by which POQ affects intensive smartphone use from the perspective of the P-E fit, other mechanisms may also exist (e.g., self-efficacy and relative deprivation), which require further examinations. Meanwhile, this study regards affective commitment as a situational factor to alleviate job boredom of employees. However, we did not further explore the mechanism by which affective commitment affects job boredom. Previous studies have shown that employees with high affective commitment could carry out more job crafting (Naeem et al., 2021), which, in turn, reduces job boredom (Harju et al., 2016). Future research could further explore the mediating mechanism between affective commitment and job boredom. Moreover, as we conceptualized affective commitment as a person-organization fit in this research, future studies could also consider the impact of the other three types of fit on the effect of POQ (person-job misfit).

Second, despite that our data were nested, we analyzed the data at the individual level while controlling the variation at the team level. It is worth noting that the use of smartphones may also be related to the characteristics of the work team or even the organization. Future research could perform multilevel analysis using nested data to explore the impact of the team or organizational factors on intensive smartphone use. Moreover, we collected data over multiple periods, but reverse causality is still possible. Thus, future research is supposed to apply a longitudinal design or experimental design to get a stronger causal relationship between POQ and intensive smartphone use.

## Conclusion

In brief, we identified a critical insufficiency in the area of intensive smartphone use, which is the lack of a P-E fit perspective in the antecedent investigation. Therefore, this research addresses the above issue and draws on the P-E fit theory (Edwards, 2008; van Vianen, 2018) to investigate the mechanism and boundary conditions in the relationship between POQ, a typical person-job fit, and intensive smartphone use. The findings demonstrated that job boredom mediates the relationship between POQ and intensive smartphone use, and the mediation path was weaker for employees with higher affective commitment. The findings of this study contribute to a more comprehensive understanding of the emergence of intensive smartphone use from a P-E fit perspective.

## Data Availability Statement

The raw data supporting the conclusions of this article will be made available by the authors, without undue reservation.

## Ethics Statement

The studies involving human participants were reviewed and approved by IRB of the School of Labor and Human Resources, Renmin University of China. The patients/participants provided their written informed consent to participate in this study.

## Author Contributions

XP: conceptualization, data collection and analysis, and drafting. KY: conceptualization, drafting, and revising the manuscript. KZ, HX, and JP: drafting and validating the final submitted version. All authors contributed to the article and approved the submitted version.

## Conflict of Interest

The authors declare that the research was conducted in the absence of any commercial or financial relationships that could be construed as a potential conflict of interest.

## Publisher’s Note

All claims expressed in this article are solely those of the authors and do not necessarily represent those of their affiliated organizations, or those of the publisher, the editors and the reviewers. Any product that may be evaluated in this article, or claim that may be made by its manufacturer, is not guaranteed or endorsed by the publisher.

## References

[B1] Aiken L. S. West S. G. (1991). *Multiple Regression: Testing and Interpreting Interactions.* Thousand Oaks, CA: Sage.

[B2] Barnes S. J. Pressey A. D. Scornavacca E. (2019). Mobile ubiquity: understanding the relationship between cognitive absorption, smartphone addiction and social network services. *Comput. Hum. Behav.* 90 246–258. 10.1016/j.chb.2018.09.013

[B3] Berger S. Wyss A. M. Knoch D. (2018). Low self-control capacity is associated with immediate responses to smartphone signals. *Comput. Hum. Behav.* 86 45–51. 10.1016/j.chb.2018.04.031

[B4] Brislin R. W. (1980). “Translation and content analysis of oral and written materials,” in *Handbook of Cross-Cultural Psychology*, Vol. 2 eds Triandis H. C. Berry J. W. (Boston, MA: Allyn & Bacon), 349–444. 10.3390/healthcare6030093

[B5] Busch P. A. McCarthy S. (2021). Antecedents and consequences of problematic smartphone use: a systematic literature review of an emerging research area. *Comput. Hum. Behav.* 114 106414. 10.1016/j.chb.2020.106414

[B6] Chen C. Zhang K. Z. K. Gong X. Zhao S. J. Lee M. K. O. Liang L. (2017). Examining the effects of motives and gender differences on smartphone addiction. *Comput. Hum. Behav.* 75 891–902. 10.1016/j.chb.2017.07.002

[B7] Chen H. Wang H. Yuan M. Xu S. (2021). Daily challenge/hindrance demands and cognitive wellbeing: a multilevel moderated mediation model. *Front. Psychol.* 12:616002. 10.3389/fpsyg.2021.616002 33762996 PMC7982414

[B8] Cheng B. Zhou X. Guo G. Yang K. (2020). Perceived overqualification and cyberloafing: a moderated-mediation model based on equity theory. *J. Bus. Ethics* 164 565–577. 10.1007/s10551-018-4026-8

[B9] Chiu S. I. (2014). The relationship between life stress and smartphone addiction on Taiwanese university student: a mediation model of learning self-efficacy and social self-efficacy. *Comput. Hum. Behav.* 34 49–57. 10.1016/j.chb.2014.01.024

[B10] David M. E. Roberts J. A. Christenson B. (2018). Too much of a good thing: investigating the association between actual smartphone use and individual well-being. *Int. J. Hum. Comput. Interact.* 34 265–275. 10.1080/10447318.2017.1349250

[B11] Edwards J. R. (2008). Person–environment fit in organizations: an assessment of theoretical progress. *Acad. Manage. Ann.* 2 167–230. 10.5465/19416520802211503

[B12] Edwards J. R. Lambert L. S. (2007). Methods for integrating moderation and mediation: a general analytical framework using moderated path analysis. *Psychol. Methods* 12 1–22. 10.1037/1082-989X.12.1.1 17402809

[B13] Edwards J. R. Van Harrison R. (1993). Job demands and worker health: three-dimensional reexamination of the relationship between person-environment fit and strain. *J. Appl. Psychol.* 78 628–648. 10.1037/0021-9010.78.4.628 8407706

[B14] Elhai J. D. Vasquez J. K. Lustgarten S. D. Levine J. C. Hall B. J. (2018). Proneness to boredom mediates relationships between problematic smartphone use with depression and anxiety severity. *Soc. Sci. Comput. Rev.* 36 707–720. 10.1177/0894439317741087

[B15] Enders C. K. Tofighi D. (2007). Centering predictor variables in cross-sectional multilevel models: a new look at an old issue. *Psychol. Methods* 12 121–138. 10.1037/1082-989X.12.2.121 17563168

[B16] Erdogan B. Bauer T. N. (2009). Perceived overqualification and its outcomes: the moderating role of empowerment. *J. Appl. Psychol.* 94 557–565. 10.1037/a0013528 19271809

[B17] Erdogan B. Karaeminogullari A. Bauer T. N. Ellis A. M. (2020). Perceived overqualification at work: implications for extra-role behaviors and advice network centrality. *J. Manage.* 46 583–606. 10.1177/0149206318804331

[B18] Feldman D. C. (1996). The nature, antecedents and consequences of underemployment. *J. Manage.* 22 385–407. 10.1177/014920639602200302

[B19] Fullwood C. Quinn S. Kaye L. K. Redding C. (2017). My virtual friend: a qualitative analysis of the attitudes and experiences of smartphone users: implications for smartphone attachment. *Comput. Hum. Behav.* 75 347–355. 10.1016/j.chb.2017.05.029

[B20] Green M. Kovacova M. Valaskova K. (2020). Smartphone addiction risk, depression psychopathology, and social anxiety. *Anal. Metaphys.* 19 52–58. 10.22381/AM1920205

[B21] Hall R. J. Snell A. F. Foust M. S. (1999). Item parceling strategies in SEM: investigating the subtle effects of unmodeled secondary constructs. *Organ. Res. Methods* 2 233–256. 10.1177/109442819923002

[B22] Harari M. B. Manapragada A. Viswesvaran C. (2017). Who thinks they’re a big fish in a small pond and why does it matter? A meta-analysis of perceived overqualification. *J. Vocat. Behav.* 102 28–47. 10.1016/j.jvb.2017.06.002

[B23] Harju L. K. Hakanen J. J. (2016). An employee who was not there: a study of job boredom in white-collar work. *Pers. Rev.* 45 374–391. 10.1108/PR-05-2015-0125

[B24] Harju L. K. Hakanen J. J. Schaufeli W. B. (2016). Can job crafting reduce job boredom and increase work engagement? A three-year cross-lagged panel study. *J. Vocat. Behav.* 95–96 11–20. 10.1016/j.jvb.2016.07.001

[B25] Harju L. K. Schaufeli W. B. Hakanen J. J. (2018). A multilevel study on servant leadership, job boredom and job crafting. *J. Manage. Psychol.* 33 2–14. 10.1108/JMP-08-2016-0237

[B26] Harold C. M. Oh I.-S. Holtz B. C. Han S. Giacalone R. A. (2016). Fit and frustration as drivers of targeted counterproductive work behaviors: a multifoci perspective. *J. Appl. Psychol.* 101 1513–1535. 10.1037/apl0000150 27504662

[B27] Hartanto A. Yang H. (2016). Is the smartphone a smart choice? The effect of smartphone separation on executive functions. *Comput. Hum. Behav.* 64 329–336. 10.1016/j.chb.2016.07.002

[B28] Harwood J. Dooley J. J. Scott A. J. Joiner R. (2014). Constantly connected-the effects of smart-devices on mental health. *Comput. Hum. Behav.* 34 267–272. 10.1016/j.chb.2014.02.006

[B29] Hofmann D. A. (1997). An overview of the logic and rationale of hierarchical linear models. *J. Manage.* 23 723–744. 10.1177/014920639702300602

[B30] Horwood S. Anglim J. (2019). Problematic smartphone usage and subjective and psychological well-being. *Comput. Hum. Behav.* 97 44–50. 10.1016/j.chb.2019.02.028

[B31] Hu J. Erdogan B. Bauer T. N. Jiang K. F. Liu S. B. Li Y. H. (2015). There are lots of big fish in this pond: the role of peer overqualification on task significance, perceived fit, and performance for overqualified employees. *J. Appl. Psychol.* 100 1228–1238. 10.1037/apl0000008 25546266

[B32] Jansen K. J. Kristof-Brown A. (2006). Toward a multidimensional theory of person-environment fit. *J. Manage. Issues* 18 193–212. 10.2307/40604534

[B33] Kim J. Park J. Sohn Y. W. Lim J. I. (2021). Perceived overqualification, boredom, and extra-role behaviors: testing a moderated mediation model. *J. Career Dev.* 48 400–414. 10.1177/0894845319853879

[B34] Kliestik T. Scott J. Musa H. Suler P. (2020). Addictive smartphone behavior, anxiety symptom severity, and depressive stress. *Anal. Metaphys.* 19 45–51. 10.22381/AM1920204

[B35] Kristof-Brown A. L. Jansen K. J. Colbert A. E. (2002). A policy-capturing study of the simultaneous effects of fit with jobs, groups, and organizations. *J. Appl. Psychol.* 87 985–993. 10.1037/0021-9010.87.5.985 12395823

[B36] Kristof-Brown A. L. Zimmerman R. D. Johnson E. C. (2005). Consequences of individuals’ fit at work: a meta-analysis of person-job, person-organization, person-group, and person-supervisor fit. *Pers. Psychol.* 58 281–342. 10.1111/j.1744-6570.2005.00672.x

[B37] Kwon H. E. So H. Han S. P. Oh W. (2016). Excessive dependence on mobile social apps: a rational addiction perspective. *Inf. Syst. Res.* 27 919–939. 10.1287/isre.2016.0658 19642375

[B38] Lapointe É Vandenberghe C. (2017). Supervisory mentoring and employee affective commitment and turnover: the critical role of contextual factors. *J. Vocat. Behav.* 98 98–107. 10.1016/j.jvb.2016.10.004

[B39] Lǎzǎroiu G. Kovacova M. Siekelova A. Vrbka J. (2020). Addictive behavior of problematic smartphone users: the relationship between depression, anxiety, and stress. *Rev. Contemp. Philos.* 19 50–56. 10.22381/RCP1920204

[B40] Li C. S. Liao H. Han Y. (2021). I despise but also envy you: a dyadic investigation of perceived overqualification, perceived relative qualification, and knowledge hiding. *Pers. Psychol.* 10.1111/peps.12444 [Epub ahead of print].

[B41] Li L. Lin T. T. C. (2018). Examining how dependence on smartphones at work relates to Chinese employees’ workplace social capital, job performance, and smartphone addiction. *Inf. Dev.* 34 489–503. 10.1177/0266666917721735

[B42] Lin C.-P. (2010). Learning virtual community loyalty behavior from a perspective of social cognitive theory. *Int. J. Hum. Comput. Interact.* 26 345–360. 10.1080/10447310903575481

[B43] Liu Q. Q. Zhang D. J. Yang X. J. Zhang C. Y. Fan C. Y. Zhou Z. K. (2018). Perceived stress and mobile phone addiction in Chinese adolescents: a moderated mediation model. *Comput. Hum. Behav.* 87 247–253. 10.1016/j.chb.2018.06.006

[B44] Liu S. Q. Luksyte A. Zhou L. Shi J. Q. Wang M. (2015). Overqualification and counterproductive work behaviors: examining a moderated mediation model. *J. Organ. Behav.* 36 250–271. 10.1002/job.1979

[B45] Liu S. Q. Wang M. (2012). Perceived overqualification: a review and recommendations for research and practice. *Res. Occup. Stress Wellbeing* 10 1–42. 10.1108/s1479-355520120000010005

[B46] Luksyte A. Bauer T. N. Debus M. E. Erdogan B. Wu C. H. (2020). Perceived overqualification and collectivism orientation: implications for work and nonwork outcomes. *J. Manage.* 48 319–349. 10.1177/0149206320948602

[B47] Luksyte A. Spitzmueller C. (2016). When are overqualified employees creative? It depends on contextual factors. *J. Organ. Behav.* 37 635–653. 10.1002/job.2054

[B48] Maynard D. C. Joseph T. A. Maynard A. M. (2006). Underemployment, job attitudes, and turnover intentions. *J. Organ. Behav.* 27 509–536. 10.1002/job.389

[B49] Meyer J. P. Allen N. J. Smith C. A. (1993). Commitment to organizations and occupations: extension and test of a three-component conceptualization. *J. Appl. Psychol.* 78 538–551. 10.1037/0021-9010.78.4.538

[B50] Mikulas W. L. Vodanovich S. J. (1993). The essence of boredom. *Psychol. Rec.* 43 3–12.

[B51] Naeem R. M. Channa K. A. Hameed Z. Ali Arain G. Islam Z. U. (2021). The future of your job represents your future: a moderated mediation model of transformational leadership and job crafting. *Pers. Rev.* 50 207–224. 10.1108/PR-07-2019-0404

[B52] Nasser F. Wisenbaker J. (2003). A Monte Carlo study investigating the impact of item parceling on measures of fit in confirmatory factor analysis. *Educ. Psychol. Meas.* 63 729–757. 10.1177/0013164403258228

[B53] Oh I. S. Guay R. P. Kim K. Harold C. M. Lee J. H. Heo C. G. (2014). Fit happens globally: a meta-analytic comparison of the relationships of person-environment fit dimensions with work attitudes and performance across East Asia, Europe, and North America. *Pers. Psychol.* 67 99–152. 10.1111/peps.12026

[B54] Podsakoff P. M. MacKenzie S. B. Lee J. Y. Podsakoff N. P. (2003). Common method biases in behavioral research: a critical review of the literature and recommended remedies. *J. Appl. Psychol.* 88 879–903. 10.1037/0021-9010.88.5.879 14516251

[B55] Porter T. Potcovaru A.-M. Zauskova A. Rowland Z. Grupac M. (2020). Smartphone addiction risk, anxiety symptom severity, and depression psychopathology. *Rev. Contemp. Philos.* 19 57–63. 10.22381/RCP1920205

[B56] Reijseger G. Schaufeli W. B. Peeters M. C. W. Taris T. W. van Beek I. Ouweneel E. (2013). Watching the paint dry at work: psychometric examination of the Dutch boredom scale. *Anxiety Stress Coping* 26 508–525. 10.1080/10615806.2012.720676 22998116

[B57] Roberts J. A. Pullig C. Manolis C. (2015). I need my smartphone: a hierarchical model of personality and cell-phone addiction. *Pers. Individ. Differ.* 79 13–19. 10.1016/j.paid.2015.01.049

[B58] Rodrigues F. R. Pina e Cunha M. Castanheira F. Bal P. M. Jansen P. G. W. (2020). Person-job fit across the work lifespan – the case of classical ballet dancers. *J. Vocat. Behav.* 118:103400. 10.1016/j.jvb.2020.103400

[B59] Sánchez-Cardona I. Vera M. Martínez-Lugo M. Rodríguez-Montalbán R. Marrero-Centeno J. (2020). When the job does not fit: the moderating role of job crafting and meaningful work in the relation between employees’ perceived overqualification and job boredom. *J. Career Assess.* 28 257–276. 10.1177/1069072719857174

[B60] Schreurs B. Hamstra M. R. Jawahar I. M. Akkermans J. (2020). Perceived overqualification and counterproductive work behavior: testing the mediating role of relative deprivation and the moderating role of ambition. *Pers. Rev.* 50 1038–1055. 10.1108/PR-05-2019-0237

[B61] Scott J. Pera A. Valaskova K. Horak J. Durana P. (2020). Problematic smartphone use severity: behavioral addiction, psychiatric symptoms, and pathological personality traits. *Rev. Contemp. Philos.* 19 64–70. 10.22381/RCP1920206

[B62] Servidio R. (2019). Self-control and problematic smartphone use among Italian university students: the mediating role of the fear of missing out and of smartphone use patterns. *Curr. Psychol.* 40 4101–4111. 10.1007/s12144-019-00373-z

[B63] Smetaniuk P. (2014). A preliminary investigation into the prevalence and prediction of problematic cell phone use. *J. Behav. Addict.* 3 41–53. 10.1556/JBA.3.2014.004 25215213 PMC4117273

[B64] Spagnoli P. Balducci C. Fabbri M. Molinaro D. Barbato G. (2019). Workaholism, intensive smartphone use, and the sleep-wake cycle: a multiple mediation analysis. *Int. J. Environ. Res. Public Health* 16:3517. 10.3390/ijerph16193517 31547191 PMC6801767

[B65] Stinglhamber F. Vandenberghe C. (2003). Organizations and supervisors as sources of support and targets of commitment: a longitudinal study. *J. Organ. Behav.* 24 251–270. 10.1002/job.192

[B66] Taylor P. Kral P. Vrbka J. Gregova E. (2020). Problematic smartphone use, social anxiety symptom severity, and technology-related behaviors and attitudes. *Anal. Metaphys.* 19 73–79. 10.22381/AM1920208

[B67] Taylor S. G. Bedeian A. G. Kluemper D. H. (2012). Linking workplace incivility to citizenship performance: the combined effects of affective commitment and conscientiousness. *J. Organ. Behav.* 33 878–893. 10.1002/job.773

[B68] Toscanelli C. Udayar S. Urbanaviciute I. Massoudi K. (2021). The role of individual characteristics and working conditions in understanding boredom at work. *Pers. Rev.* 10.1108/PR-07-2020-0510 [Epub ahead of print].

[B69] van Hooff M. L. M. van Hooft E. A. J. (2014). Boredom at work: proximal and distal consequences of affective work-related boredom. *J. Occup. Health Psychol.* 19 348–359. 10.1037/a0036821 24885686

[B70] van Vianen A. E. M. (2018). Person–environment fit: a review of its basic tenets. *Annu. Rev. Organ. Psychol. Organ. Behav.* 5 75–101. 10.1146/annurev-orgpsych-032117-104702

[B71] Vandenberghe C. Bentein K. Panaccio A. (2017). Affective commitment to organizations and supervisors and turnover: a role theory perspective. *J. Manage.* 43 2090–2117. 10.1177/0149206314559779

[B72] Wang Y. Yang H. Montag C. Elhai J. D. (2020). Boredom proneness and rumination mediate relationships between depression and anxiety with problematic smartphone use severity. *Curr. Psychol.* 10.1007/s12144-020-01052-0 [Epub ahead of print].

[B73] Wayne J. H. Casper W. J. Matthews R. A. Allen T. D. (2013). Family-supportive organization perceptions and organizational commitment: the mediating role of work-family conflict and enrichment and partner attitudes. *J. Appl. Psychol.* 98 606–622. 10.1037/a0032491 23565896

[B74] Wu I. R. Chi N. W. (2020). The journey to leave: understanding the roles of perceived ease of movement, proactive personality, and person-organization fit in overqualified employees’ job searching process. *J. Organ. Behav.* 41 851–870. 10.1002/job.2470

[B75] Yu K. Y. T. Davis H. M. (2016). Autonomy’s impact on newcomer proactive behaviour and socialization: a needs-supplies fit perspective. *J. Occup. Organ. Psychol.* 89 172–197. 10.1111/joop.12116

[B76] Zhang F. F. Wang B. Qian J. Parker S. K. (2021a). Job crafting towards strengths and job crafting towards interests in overqualified employees: different outcomes and boundary effects. *J. Organ. Behav.* 42 587–603. 10.1002/job.2517

[B77] Zhang M. Wang F. Li N. (2021b). The effect of perceived overqualification on creative performance: person-organization fit perspective. *Front. Psychol.* 12:582367. 10.3389/fpsyg.2021.582367 34054629 PMC8155303

[B78] Zhang M. Wang F. Weng H. L. Zhu T. Liu H. Y. (2021c). Transformational leadership and perceived overqualification: a career development perspective. *Front. Psychol.* 12:597821. 10.3389/fpsyg.2021.597821 33643130 PMC7904678

[B79] Zhang M. J. Law K. S. Lin B. (2016). You think you are big fish in a small pond? Perceived overqualification, goal orientations, and proactivity at work. *J. Organ. Behav.* 37 61–84. 10.1002/job.2024

